# Vehiculation of Methyl Salicylate from Microcapsules Supported on Textile Matrix

**DOI:** 10.3390/ma14051087

**Published:** 2021-02-26

**Authors:** Samira Mendes, André Catarino, Andréa Zille, Nádia Fernandes, Fabricio Maestá Bezerra

**Affiliations:** 1 Textile Engineering, Federal University of Technology—Paraná, 635 Marcílio Dias St, Apucarana, PR 86812-460, Brazil; fabriciom@utfpr.edu.br; 2 2C2T—Center for Textile Science and Technology, University of Minho, Campus de Azurém, 4800-058 Guimarães, Portugal; whiteman@det.uminho.pt (A.C.); azille@det.uminho.pt (A.Z.); 3 Department of Chemical Engineering, State University of Maringá, Colombo Avenue, Maringá, PR 5790, Brazil; nadiareginacfm@hotmail.com

**Keywords:** textile, microcapsules, controlled release

## Abstract

In recent years, textile industries have focused their attention on the development of functional finishing that presents durability and, consequently, controlled release. However, in the case of methyl salicylate microcapsules supported on a textile matrix, studies indicate only the interactions between substrate and microcapsules and the drug delivery system, not applying the release equations. This study reports the mechanism and kinetics of controlled release of microcapsules of gelatin and gum Arabic containing methyl salicylate as active ingredient incorporated into textile matrices. According to the results presented, it was possible to verify that the wall materials participated in the coacervation process, resulting in microcapsules with well-defined geometry, besides promoting the increase of the thermal stability of the active principle. The samples (100% cotton, CO, and 100% polyamide, PA) functionalized with microcapsules released methyl salicylate in a controlled manner, based on the adjustment made by the Korsmeyer–Peppas model, indicating a Fickian mechanism. The influence of temperature was noticeable when the samples were subjected to washing, since with higher temperature (50 °C), the release was more pronounced than when subjected to lower temperature (37 °C). The results presented in this study indicate that the mechanism of backbone release is influenced by the textile matrix and by the durability of the microcapsule during the wash cycles.

## 1. Introduction

Drugs, essential oils and fragrances have been used as essential compounds in textile finishing, in order to meet the various requirements that the consumer presents today. Many of these chemical compounds are highly volatile, which may present a disadvantage in their application on the surface of the fabric, due to their rapid and uncontrolled release. Therefore, one of the main focuses of the textile industry is the production of finishing that has durability, that is, release the active ingredient in a controlled way, consequently leading to an increase in its life cycle [1].

Microencapsulation aims to coat active agents, protecting them from adverse environmental conditions, such as light, humidity, oxygen and other compounds, which could result in degradation or polymerization of the active agent [2,3]. There are several microencapsulation techniques that can be used to improve the stability of the active ingredients, such as simple or complex coacervation, fluidization, lyophilization and spray drying [4,5]. Among these techniques, complex coacervation is an alternative process, in which the ionic interaction between two polymers leads to the formation of coacervates and phase separation [6]. Such a technique can produce microcapsules applicable to the textile industry, due to the fact that they have resistance to temperature and have favorable characteristics for controlled release [7,8].

Some studies show the application of microcapsules in textile articles, as in the work of Lis et al. [9], Bezerra et al. [10] and Carreras et al. [11], indicating that microcapsules can be used as fabric finishing, increasing their functionality. The fabrics that become supports for the release of active ingredients are called biofunctional textiles. These can be defined as textile substrates that have been modified to attain new properties and added value [9]. Some examples of biofunctional textiles are antimicrobial and cosmetotextiles [12].

The advantages of using biofunctional textiles are a high area of contact with the skin, drug loading capacity, ease of application, low price, release by stimulation, biocompatibility and being non-allergic and non-toxic, among other properties [13,14,15]. Thus, the choice of the active principle is of importance to the desired effect of the fabric.

Methyl salicylate is a chemical compound widely used for treatments of musculoskeletal pain, such as athletic injuries, swelling, tension or torsion [16,17]. Generally, the treatment involves external application to the affected area of a drug that has methyl salicylate as its active ingredient. However, it has some drawbacks, such as low water solubility and dosage control, as well as high volatility [18,19,20]. This means that the concentration of methyl salicylate should be controlled to ensure effective delivery of the drug, in order to achieve anti-inflammatory and analgesic effects and, on the other hand, to ensure that there is no risk of skin irritation or burns [17,20,21,22]. Yang et al. [19] carried out a microencapsulation study in which they used chitosan as a wall material, having observed that the microencapsulation process provided a controlled release when compared to free methyl salicylate.

In this context, the present study aims to evaluate the behavior of methyl salicylate microcapsules obtained by complex coacervation and incorporated in Jersey 100% cotton and 100% polyamide textile structures, using in vitro release profiles, and when subjected to washing at different temperatures.

## 2. Materials and Methods

The materials used for the development of the microcapsules were gelatin and gum Arabic (Sigma-Aldrich, São Paulo, SP, Brazil), methyl salicylate (Induslab, Londrina, PR, Brazil) as well as the sodium lauryl sulfate (Exodos, Sumaré, SP, Brazil). For the microcapsules cross-linking, glutaraldehyde (25%) (Sigma-Aldrich, São Paulo, SP, Brazil). The materials used in the esterification of the finishing were citric acid (Synth, Diadema, SP, Brazil) and sodium hypophosphite (SHIP) (Sigma-Aldrich, São Paulo, SP, Brazil). 

Jersey knits were used in the compositions of 100% cotton, with a weight of 280 g m^−2^, and 100% polyamide, with a weight of 200 g m^−2^, both produced at the Process Research Laboratory of the University of Minho, Guimarães, Portugal.

### 2.1. Preparation of Microcapsules

The preparation of the microcapsules was performed through the complex coacervation method based on the techniques presented in the works of Yang et al. [23], Butstraen and Salaün [24] and Bezerra et al. [25], which presented the use of biopolymers, pH adjustment and active principle.

Three different solutions were prepared, which used deionized water as a solvent, and placed in a thermostated bath at 40 °C. The first solution was constituted by dissolving 3 g of gelatin in 50 mL of solution, subjected to magnetic stirring at 300 rpm for 15 min. The second emulsion was composed of 0.3 g of sodium lauryl sulfate and 3 mL of methyl salicylate in 50 mL of solution, under stirring at 300 rpm for 15 min. Finally, the third solution was prepared by inserting 3 g of gum Arabic into 100 mL of water, under magnetic stirring at 300 rpm for 15 min. Subsequently, the methyl salicylate solution was added dropwise to the gelatin solution to form a colloidal emulsion system with the active ingredient, which was mechanically stirred at 500 rpm for 10 min. The insertion of the third solution was also performed dropwise, in order to ensure a slow interaction between the biopolymers. Stirring occurred at 700 rpm for 10 min. In order to ensure the reaction between gelatin and gum Arabic, positive and negative polyelectrolyte, the pH was adjusted with 5 mol L^−1^ citric acid to pH 4.1, which fits into what was discussed by Duhoranimana et al. [26] as the best interaction range, namely pH 4.0–4.5. The solution was stirred for 90 min.

For the gelation process, the solution was cooled to a range of 0 to 8 °C and kept under stirring for 60 min. After this process, the microcapsules were cross-linked with 0.5 g glutaraldehyde (25%), which was added to the solution after it was adjusted to pH 8–9 with NaOH 1 mol L^−1^ and kept under stirring at 700 rpm for 30 min. 

The microcapsules were kept in solution to promote greater impregnation of methyl salicylate in the textile substrates, as the active ingredient that has not been encapsulated may also be deposited on the surface of the textile article.

### 2.2. Characterization of the Microcapsules

The interaction of the polymers for the production of microcapsules was evaluated by FTIR (Nicolet Avatar, Golden Valey, MN, USA), in the range of 4000 to 500 cm^−1^. For thermal stability (TG), the test was performed at a speed of 10 °C min^−1^, in the temperature range of 30 °C to 800 °C in a nitrogen atmosphere with a flow of 50 mL min^−1^ (Shimadzu, model TG-50,Tokyo, Japan). The morphology and structure of the microcapsules were evaluated by SEM (Quanta 250, Hillsboro, OR, USA).

### 2.3. Functionalization of Textile Substrates

The functionalization of the textile substrates occurred by esterification with citric acid and application of the microcapsules. The application of microcapsules was performed through the pad-dry-cure process, followed by a drying process at 80 °C for 3 min and, subsequently, the cure was performed at 120 °C for 2 min, a technique that has been adapted from the work of Rodrigues et al. [27], Azizi et al. [28] and Nada et al. [29]. 

The textile articles, cotton and polyamide, were impregnated for 1 min in a solution of microcapsules (30 g $L^{-1}$), citric acid (30 g $L^{-1}$) and sodium hypophosphite (10 g $L^{-1}$) at 25 °C and pH 6, and subsequently, the samples were taken to the foulard process. The pick-up adopted was 80%.

### 2.4. Evaluation of the Functionalization of Textile Substrates 

To evaluate the chemical modification on the surface of the textile substrates after the functionalization with microcapsules, this study used the Fourier transform infrared spectroscopy technique in the infrared region with attenuated reflectance (FTIR/ATR), in the range between 4000 to 600 cm^−1^. The surface of the textile articles, untreated and treated with microcapsules, was analyzed by scanning electron microscopy.

The study of the in vitro controlled release of methyl salicylate from cotton and polyamide substrates was performed in triplicate. The functionalized samples were sent to a water and ethanol (2:1 v/v) bath thermostated at 37 °C under stirring in a shaker (Solab, Piracicaba, SP, Brazil). At predetermined times, aliquots were taken and filtered, to determine the absorbances in the wavelength of methyl salicylate at 306 nm, by the UV–Vis technique (Shimadzu, Tokyo, Japan).

The functionalized textile samples were subjected to the washing test with procedure adapted from the Standard AATCC 61-2007-2A (Colorfastness to Laundering: Accelerated). They were inserted into mugs (Kimak, Brusque, SC, Brazil) under the following conditions: 50 mL of solution with steel balls for 45 min, the temperature varying between 37 and 50 °C. At each wash cycle, aliquots were taken and filtered to determine the amount of methyl salicylate by absorbance in the spectrophotometer in the ultraviolet region (UV–Vis).

## 3. Results and Discussion

### 3.1. Evaluation of the Functional Groups of the Microcapsules (FTIR)

**Figure 1:**
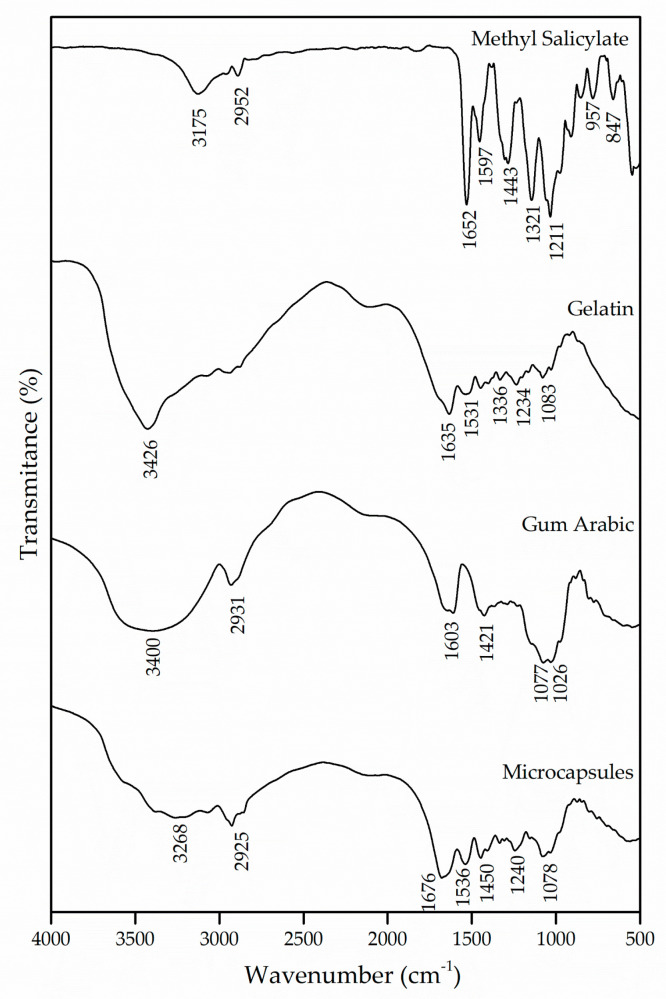
Infrared spectroscopy (FTIR) of methyl salicylate, gelatin and gum Arabic and the microcapsules obtained.

The interactions of the polymers used for the formation of the microcapsules were evaluated by the FTIR technique. Figure 1 shows the spectrograms of the microcapsules that were obtained, as well as those of the wall materials, gelatin and gum Arabic and of the active ingredient methyl salicylate.

Methyl salicylate has in its molecule intramolecular hydrogen bonds between hydroxyl and carbonyl groups [20]. The bands in the regions of 3175 cm^−1^ and 2952 cm^−1^ are attributed to the hydroxyl group and the asymmetrical stretching of the CH_2_, respectively. The band at 1652 cm^−1^ was derived from the existence of C=O stretching vibrations of the carboxylic acid group [30]. Likewise, the bands in the regions of 1597 cm^−1^ and 1443 cm^−1^ appear due to the asymmetrical and symmetrical stretching of the deprotonated carboxylate group, respectively [31].

The gelatin, being a protein, is characterized by amide and amine groups in its chemical structure. The absorption band in the region of 3426 cm^−1^ corresponds to the vibrations of the amino functional groups N–H [29,32,33]. The C=O stretching of peptide bonds and the deformation of the N–H were identified in the band of 1635 cm^−1^, which refers to the region of the amide I [34,35,36]. Another absorption characteristic of the gelatin occurs in the region of 1531 cm^−1^, which can be attributed to the amide II due to the flexion of the groups N–H and the stretching of C–N [36,37]. The absorption of the band in the region of 1234 cm^−1^ is caused by the stretching vibration of the groups N–H and C–N, corresponding to the amide III [38].

The gum Arabic has in its chemical structure carboxylic groups, which justifies the presence of negative charges. The bands at 1603 cm^−1^ and 1421 cm^−1^ are derived from the asymmetric and symmetric stretching vibration of carboxylic acid ($-{\mathrm{COO}}^{-}$), respectively, which is attributed to the carboxylate groups of the glucuronic acid present in the gum Arabic [39,40,41,42]. To represent the elongation of the C–O bond, bands were detected in the regions of 1077 cm^−1^ and 1026 cm^−1^. Similar results were found by [40,43,44,45]. 

For the interaction of biopolymers to occur and for the formation of microcapsules, it is essential that both the protein and the polysaccharide have oppositely charged side groups that will allow interaction between them, promoting the coacervation process and the formation of amides. The spectrum of the microcapsules was compared with that of the wall materials, and a shift of the amide I and amide II bands from 1635 cm^−1^ and 1531 cm^−1^ to 1676 cm^−1^ and 1536 cm^−1^ can be observed, which, according to the authors Shaddel et al. [34], García-Saldaña et al. [35] and Shaddel et al. [46], evidences the generation of an electrostatic interaction between the positively charged amine groups of gelatin (${\mathrm{NH}}_{3}^{+}$) and the negatively charged carboxylic groups of gum Arabic (${\mathrm{COO}}^{-}$). Such interaction can also be confirmed with the emergence of the band in the region of 1450 cm^−1^, which indicates the presence of an amide, corroborating the formation of this complex [43,46]. Based on this, it can be assumed that the wall materials of the microcapsule participated in the microencapsulation process by electrostatic interaction and that hydrogen bonds were also involved in the coacervation of the biopolymers.

### 3.2. Thermal Stability (TG/dTG)

**Figure 2:**
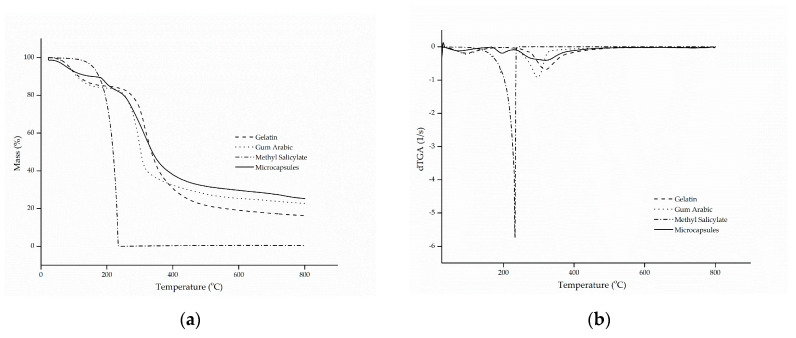
(**a**) thermal stability (TG) and (**b**) thermogravimetric curve (dTG) of gelatin, gum Arabic, methyl salicylate and microcapsules.

The thermal stability of the microcapsules was evaluated by thermogravimetric curves (TG) and the curves of the first derivative of the thermogravimetric curve (dTG) with respect to time, as shown in Figure 2a,b, respectively. 

The thermogram of methyl salicylate showed only one stage of mass loss, in a configuration similar to a parabola, which starts at approximately 100 °C until full evaporation at 218 °C, a characteristic common to oils and fragrances. This phenomenon is confirmed by the evaporation temperature of the active ingredient, which is in the range of 220 to 224 °C [47].

Gelatin and gum Arabic followed similar and characteristic thermal degradation profiles, in which two stages of decomposition were presented [7,29]. The first stage is associated with the hydrolysis of the polymer chain, that is, the release of water bound to the hydrophilic groups of the polymers [48,49], which showed a mass loss of approximately 15.9% (m/m), corresponding to the maximum peak temperature at 92 °C. The second stage represents the breakdown of the molecular chains of the protein and the polysaccharide [50,51], at temperatures of 318 and 296 °C, in that order.

As for the microcapsules, it can be observed that the behavior of thermal degradation occurred in three stages, as was also observed by Matos et al. [52]. The first degradation stage, with a maximum temperature of 60 °C, corresponded to a mass loss of 12.2% (m/m), which is related to the complete release of water linked to the structure of the microcapsules and to the evaporation of the methyl salicylate present on the surface of the microcapsules. The second mass loss corresponds to the second stage, which suffered a faster decline, and it can be explained by the rapid release of the active compound due to the high volatility of the material, together with the decomposition of the protein and the polysaccharide used as the wall material. This resulted in a significant mass reduction of approximately 42% (m/m), at a temperature in the range of 324 °C. Complete degradation of the biopolymers occurred in the third stage, at 584 °C, with a mass reduction of 38.7% (m/m) [7,22,52,53].

The results presented above confirm that the encapsulation of methyl salicylate by complex coacervation microcapsules can significantly improve the thermal stability of the active compound, suggesting that it was actually encapsulated.

### 3.3. Morphology (MEV)

**Figure 3:**
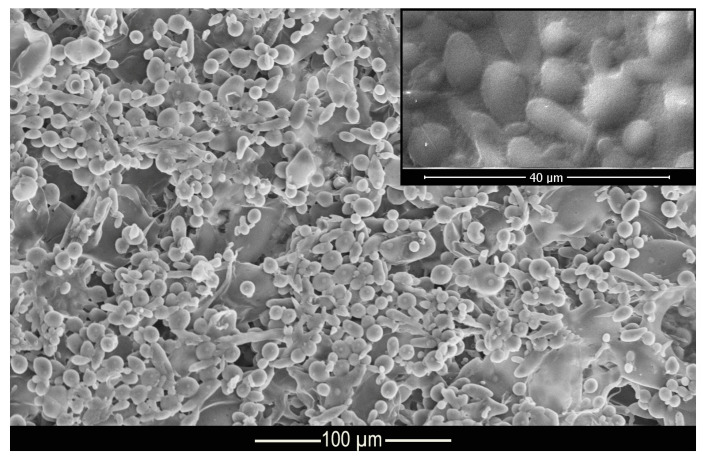
Micrograph of methyl salicylate microcapsules.

Figure 3 shows the morphology and structure of the microcapsules. According to the micrograph presented, it can be seen that the complex coacervation process formed microcapsules with well-defined geometry in the shape of elongated spheres, as shown in the Muhoza et al. [54] that used the complex coacervation technique, with distribution of the polydisperse material and with a dispersion slightly affected by agglomerates. According to Alvim and Grosso [55], glutaraldehyde cross-linked microcapsules tend to come together during the drying process, as the glutaraldehyde is heat activated, and the aldehydes not bound to amino groups can be polymerized during the drying process. However, this shape is not a problem for this work since the elongated spheres can be deposited on the fabric surface, as well as the regular spheres. The difference in surface area of the microcapsules allows some of them to be deposited in different regions of the fiber.

### 3.4. Functionalization of Textile Substrates

**Figure 4:**
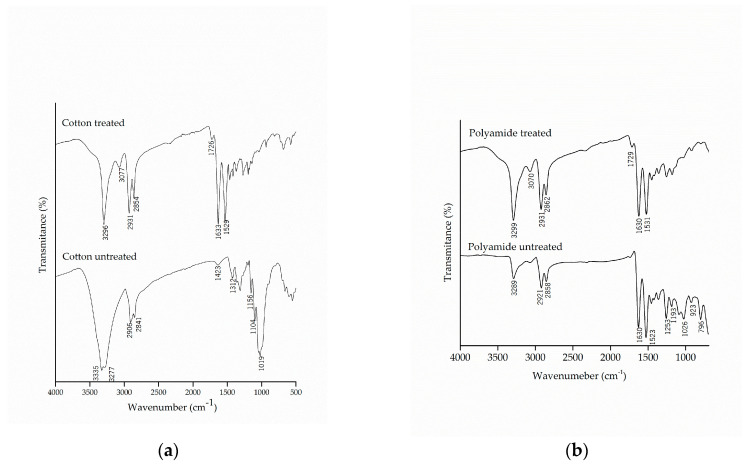
Infrared spectrum with attenuated reflectance (FTIR/ATR) of pure and treated (**a**) cotton and (**b**) polyamide substrates.

The chemical modification of the textile substrates was evaluated by FTIR-ATR, which allowed the detection of the presence of functional groups in the molecular structure of cotton and polyamide fibers, untreated and treated with the microcapsules of methyl salicylate, as shown in Figure 4.

Comparing the spectra of the untreated cotton with those from the treated samples, the appearance of the bands in the region of 1633 cm^−1^ and 1529 cm^−1^, characteristic of secondary amide, can be observed, which shows a new molecular interaction between the microcapsules and the cotton fabric. Similar results were found by Bezerra et al. [25], which verified the effectiveness of the finishing by the appearance of the band at 1540 cm^−1^ in the cotton fabric, subsequent to the incorporation of the gelatin and gum Arabic microcapsules.

It can also be noted that there was the appearance of the band at 1726 cm^−1^, which is related to the esterification between the hydroxyl groups present in the microcapsule, the carboxylic groups of citric acid and the hydroxyl group of cellulose [28,55]. The esterification reaction allows the microcapsule to incorporate into the fiber, thus making the finishing more resistant to washing, as this is one of the limiting factors for fabric finishing.

The spectra of the polyamide samples before and after the finishing are shown in Figure 4b. The treated polyamide samples did not show to be different due to the overlapping of the bands of the microcapsules with the functional groups of the polyamide. Once again, the appearance of the band in the region of 1729 cm^−1^, which represents the carbonyl group of the ester group, could be observed as a result of the chemical interaction of citric acid between the microcapsules and the polyamide.

### 3.5. Quantification and Controlled Release of Methyl Salicylate

The study of the release profile of an active compound from polymeric systems is important to understand its behavior and the mechanism by which release occurs. To better understand and evaluate the release of methyl salicylate, mathematical models were adopted to adjust the release system, and the selected models were Higuchi [56] and Korsmeyer–Peppas et al. [57].

**Figure 5:**
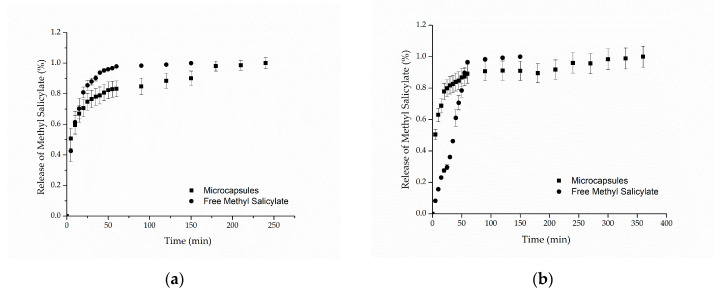
In vitro release profile of methyl salicylate from (**a**) cotton and (**b**) polyamide textile.

Figure 5a,b presents the in vitro release profiles for cotton and polyamide samples, respectively. For both samples, it can be observed that the methyl salicylate, when freely impregnated into the textile structure, was completely released in approximately 60 min, reaching 98% of the released compound. Regarding the samples functionalized with microcapsules, the results showed release profiles in two stages: a rapid initial release (burst effect), followed by a slower increase until the equilibrium, in which the maximum amount of methyl salicylate released is reached after 250 min for cotton and 350 min for polyamide. Similar behaviors were found in the works of Ma et al. [58], Aguiar et al. [59] and Macha et al. [60]. The first release step may be explained by the release of the methyl salicylate that was on the surface of the textile substrate due to the method of application, that is, the part of the active compound that was not coated by the microcapsules [60].

**Figure 6:**
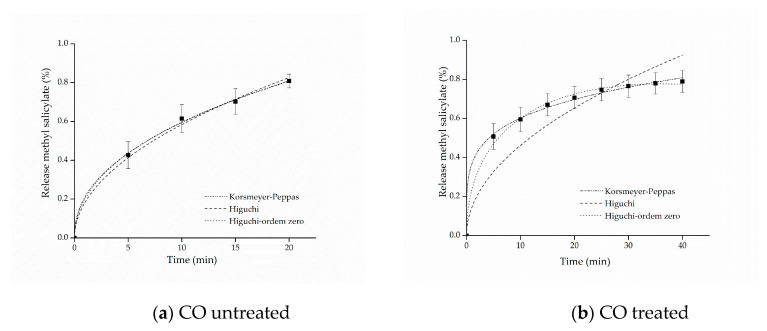
Kinetic adjustments of the profiles of controlled release from (**a**) cotton (CO) untreated and (**b**) CO treated.

**Figure 7:**
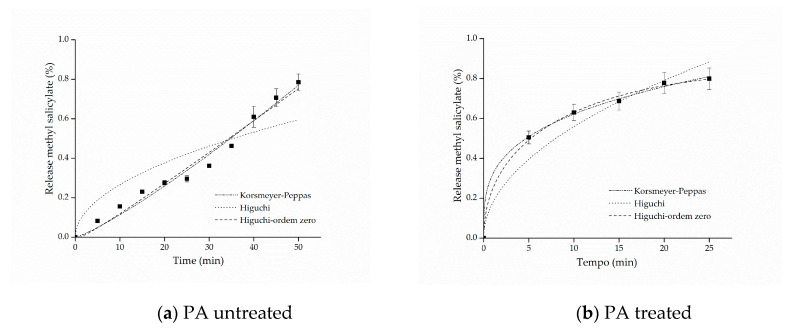
Kinetic adjustments of the profiles of controlled release from (**a**) polyamide (PA) untreated and (**b**) PA treated.

The kinetic adjustments of the profiles of controlled release from samples of cotton and polyamide, considering the first 80% of release of the active ingredient [61], are presented in Figure 6 and Figure 7.

The adjustment that best represented the release of both cotton and polyamide samples was the model proposed by Korsmeyer–Peppas. This model provides information about the release mechanism, which is evaluated using exponent *n*, as shown in Table 1. 

The parameters of the mathematical adjustments are presented in Table 2, and it is possible to observe that as regards the cotton sample with free methyl salicylate, the value of n=0.44527±0.02458 was obtained, whereas for the sample functionalized with microcapsules, the value of n=0.22322±0.00959 was obtained. According to the values of *n* presented in Table 1, both profiles indicate Fickian diffusion, caused by the degree of swelling of the matrices, which is determined by the high mobility of the polymer chains of the polymers forming the microcapsules and by the cotton fiber chains [61,62]. Other studies have also reported values of *n* lower than 0.43 for spherical polymer systems. This assumes that a reduction of this variable can be expected when the release study occurs by polydisperse systems with irregular geometries, indicating, nevertheless, the presence of Fickian mechanism [63,64].

For the polyamide sample with free salicylate, it can be observed that the value of the release exponent resulted in *n* = 1.0, indicating a zero-order release mechanism, which can be explained by the hydrophobicity of the polyamide fibers that, in contact with water, have resistance to swelling and consequently to the relaxation of polymer chains, favoring the rapid release of methyl salicylate. On the other hand, as regards the polyamide fabric treated with the microcapsules, the Korsmeyer–Peppas adjustment presented a value of *n* = 0.28872, which, according to what is shown in Table 1, is represented by the Fickian diffusion mechanism, that is, the microcapsule protects the active principle allowing the longer release time, consequently extending the life cycle of the finishing.

In this context, the release mechanisms (the value of the exponent *n*) were found according to the textile matrix, indicating that the hydrophilicity/hydrophobicity of the fabric is a determining factor of it, as reported in the works of Bezerra et al. [25] and Arias et al. [9], when they used cotton and polyester as a textile matrix to support encapsulated compounds, and the substrates influence the system release mechanism. Therefore, the mechanism can be modified according to the textile matrix, the microcapsule and the active principle, but in all experiments, their positive influence on controlled release was clear.

### 3.6. Durability of the Finishing

**Figure 8:**
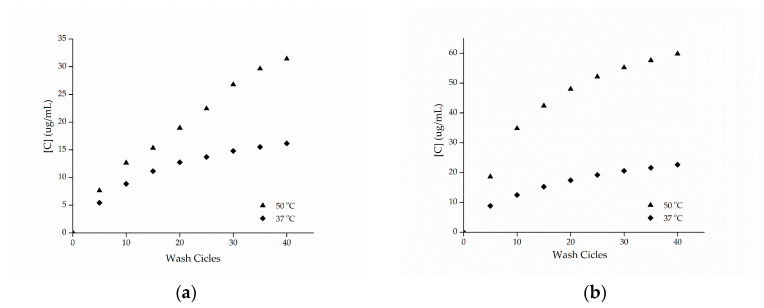
Release profile of methyl salicylate from samples of (**a**) cotton and (**b**) polyamide in the washing process.

The cotton and polyamide substrates treated with microcapsules and cross-linked with citric acid were subjected to washing tests at temperatures of 37 and 50 °C with the procedure adapted from the Standard AATCC 61-2007-2A. Figure 8 shows the release profiles of the microcapsules against the number of wash cycles. Based on these data, it can be seen that the samples presented a high resistance to washing, showing an increase in the durability of the finishing.

Cross-linking agents, such as citric acid or polycarboxylates, increase the fabric/microcapsule interaction through the esterification reaction, as shown by the FTIR-ATR technique, making the finishing more resistant to washing, as observed in the work of Bezerra et al. [10], when they used BTCA as cross-linking agent in gelatin and gum Arabic microcapsules.

Comparing the esterified samples washed at 37 and 50 °C, the influence of temperature on the release of the active ingredient can be observed, since the samples washed at a temperature of 50 °C resulting in a greater release of methyl salicylate, which can be attributed to the breakdown of the structure of the microcapsules. In the work of Sun et al. [14], the efficiency of the impregnation of porous microgels fixed on cotton fabrics via citric acid cross-linking reaction was evaluated, having been observed in the study that the release at 37 °C was higher compared to the release at 25 °C, indicating that the temperature has an effect proportional to the release of the active ingredient, as observed.

**Figure 9:**
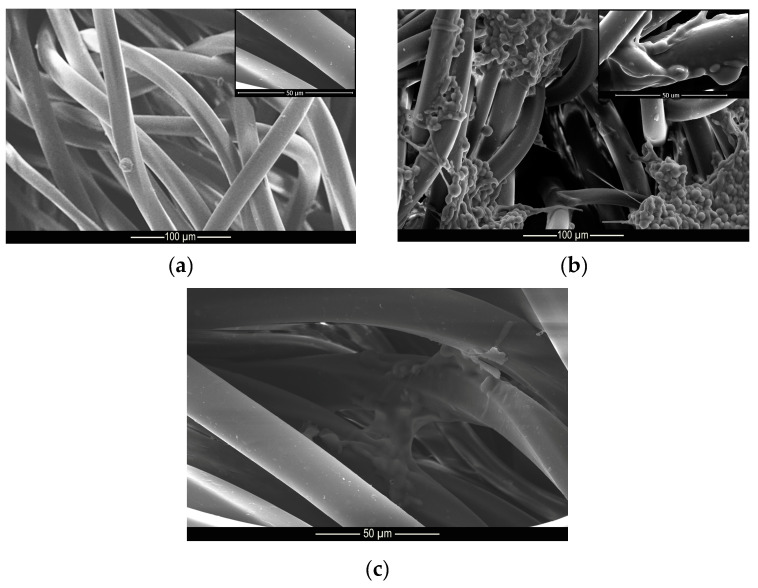
Microscopy of (**a**) untreated, (**b**) treated and (**c**) washed polyamide samples.

**Figure 10:**
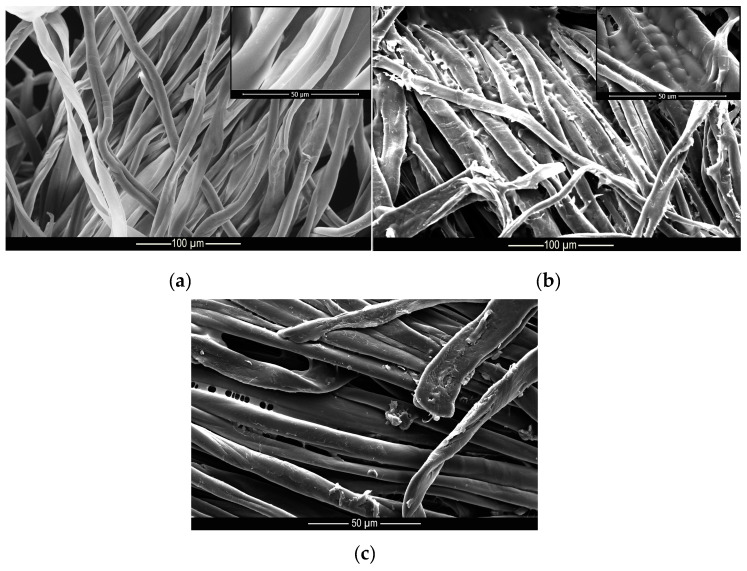
Microscopy of (**a**) untreated, (**b**) treated and (**c**) washed cotton samples.

These results indicate that the microcapsules were incorporated into the cotton and polyamide structures, since after 40 wash cycles, there were still microcapsules embedded into the substrates, as can be seen from the microscopy shown in Figure 9 and Figure 10. These results are superior to those found by Ramya et al. [65] who, when evaluating the durability to washing for herb extract microcapsules, found that the microcapsules presented good resistance up to 30 wash cycles due to the sustained release of the encapsulated extracts.

## 4. Conclusions

By observing the results presented, it can be seen that methyl salicylate microcapsules produced by complex coacervation using gelatin and gum Arabic as wall materials were obtained. The shape of the microcapsules presented a well-defined geometry and good dispersion; furthermore, the thermal stability of the active material improved significantly with the microencapsulation process, that is, the compound obtained withstands temperatures above those at which the free active ingredient resists. The microcapsules incorporated into the fabrics release the active ingredient in a controlled manner, according to the adjustment made by the Korsmeyer–Peppas model, and also presented a high resistance to washing, since the release of methyl salicylate was lower in these samples when compared to simply depositing the microcapsules. The influence of temperature was also visible since, when subjected to the washing process at higher temperatures, namely 50 °C, the release was more pronounced than at 37 °C.

In view of the aforementioned facts, the functionalization of cotton and polyamide substrates with methyl salicylate microcapsules can be considered as a promising alternative for the development of functional textile articles that will contribute to consumer welfare, due to the results of the microencapsulation and finishing durability. 

## Figures and Tables

**Table 1:** Interpretation of the release mechanisms from polymeric systems, as proposed by Korsmeyer–Peppas et al. [57].

Surface	Cylinder	Sphere	Diffusion Mechanism
0.5	0.45	0.43	Fickian
0.5 < *n* < 1.0	0.45 < *n* < 0.89	0.43 < *n* < 0.85	Anomalous
1.0	0.89	0.85	Non-Fickian

**Table 2:** Parameters of the mathematical adjustments for the models of Korsmeyer–Peppas, Higuchi and Higuchi zero-order.

Model	Parameter	CO Untreated	CO Treated
Higuchi zero-order	R^2^	0.99831	0.99507
	K_0_(10^−2^)	−0.00642 ± 0.00259	−0.02134 ± 0.00126
	K_HO_	0.2091 ± 0.01003	0.25751 ± 0.0067
Higuchi	R^2^	0.99614	0.81856
	K_H_	0.18465 ± 0.00279	0.14612 ± 0.00793
Korsmeyer–Peppas	R^2^	0.99803	0.99741
	K_KP_	0.2132 ± 0.01392	0.3687 ± 0.011287
	n	0.44527 ± 0.02458	0.2128 ± 0.01106
		**PA Untreated**	**PA Treated**
Higuchi zero-order	R^2^	0.95992	0.99712
	K_0_(10^−2^)	0.01367 ± 0.00271	−0.02178 ± 0.00228
	K_HO_	0.000066 ± 0.01441	0.26836 ± 0.00975
Higuchi	R^2^	0.83729	0.94503
	K_H_	0.07143 ± 0.00567	0.1768 ± 0.00805
Korsmeyer–Peppas	R^2^	0.96041	0.99802
	K_KP_	0.01211 ± 0.00491	0.31986 ± 0.01523
	n	1.03572 ± 0.11702	0.28872 ± 0.0171
